# Disagreement in primary study selection between systematic reviews on negative pressure wound therapy

**DOI:** 10.1186/1471-2288-8-41

**Published:** 2008-06-26

**Authors:** Frank Peinemann, Natalie McGauran, Stefan Sauerland, Stefan Lange

**Affiliations:** 1 Institute for Quality and Efficiency in Health Care (IQWiG), Dillenburger Str. 27, 51105 Cologne, Germany; 2 Institute for Research in Operative Medicine, University of Witten/Herdecke, Ostmerheimer Str. 200, 51109 Cologne, Germany

## Abstract

**Background:**

Primary study selection between systematic reviews is inconsistent, and reviews on the same topic may reach different conclusions. Our main objective was to compare systematic reviews on negative pressure wound therapy (NPWT) regarding their agreement in primary study selection.

**Methods:**

This retrospective analysis was conducted within the framework of a systematic review (a full review and a subsequent rapid report) on NPWT prepared by the Institute for Quality and Efficiency in Health Care (IQWiG).

For the IQWiG review and rapid report, 4 bibliographic databases (MEDLINE, EMBASE, The Cochrane Library, and CINAHL) were searched to identify systematic reviews and primary studies on NPWT versus conventional wound therapy in patients with acute or chronic wounds. All databases were searched from inception to December 2006.

For the present analysis, reviews on NPWT were classified as eligible systematic reviews if multiple sources were systematically searched and the search strategy was documented. To ensure comparability between reviews, only reviews published in or after December 2004 and only studies published before June 2004 were considered.

Eligible reviews were compared in respect of the methodology applied and the selection of primary studies.

**Results:**

A total of 5 systematic reviews (including the IQWiG review) and 16 primary studies were analysed. The reviews included between 4 and 13 primary studies published before June 2004. Two reviews considered only randomised controlled trials (RCTs). Three reviews considered both RCTs and non-RCTs. The overall agreement in study selection between reviews was 96% for RCTs (24 of 25 options) and 57% for non-RCTs (12 of 21 options). Due to considerable disagreement in the citation and selection of non-RCTs, we contacted the review authors for clarification (this was not initially planned); all authors or institutions responded. According to published information and the additional information provided, most differences between reviews arose from variations in inclusion criteria or inter-author study classification, as well as from different reporting styles (citation or non-citation) for excluded studies.

**Conclusion:**

The citation and selection of primary studies differ between systematic reviews on NPWT, particularly with regard to non-RCTs. Uniform methodological and reporting standards need to be applied to ensure comparability between reviews as well as the validity of their conclusions.

## Background

Although systematic reviews are a valuable tool in the synthesis of evidence, they should be interpreted with caution [1]. The sharp rise in the number of systematic reviews published over the past decades has led to a concomitant increase in discordant results and conclusions between reviews on the same research question [2-5]. This has caused disputes between researchers and created difficulties for decision-makers in selecting appropriate health care interventions. Among other things, discordance between reviews may be caused by differences in primary study selection [6] due to variations in literature search strategies, selection criteria, and the application of selection criteria [2].

The Institute for Quality and Efficiency in Health Care (Institut für Qualität und Wirtschaftlichkeit im Gesundheitswesen, IQWiG) conducted a systematic review on the effectiveness and safety of negative pressure wound therapy (NPWT) versus conventional wound therapy in patients with acute or chronic wounds. The NPWT technique aims to accelerate wound healing by placing a foam dressing in the wound and applying controlled subatmospheric pressure [7]. The German-language full review and a rapid report on studies subsequently published are available on the IQWiG website [8,9]. In addition, an English-language journal article has been published [10].

An additional retrospective analysis was conducted in order to compare different systematic reviews on NPWT regarding their agreement in primary study selection. The review methodologies were also compared.

## Methods

For the IQWiG review and rapid report, 4 bibliographic databases (MEDLINE, EMBASE, The Cochrane Library, and CINAHL) were searched to identify systematic reviews and primary studies on NPWT versus conventional wound therapy in patients with acute or chronic wounds. All databases were searched from inception to May 2005 (review) and between May 2005 and December 2006 (rapid report).

The multi-source search strategy and literature screening are described in detail elsewhere [8]. Eligible primary studies were randomised controlled trials (RCTs), as well as non-randomised controlled trials (non-RCTs) with a concurrent control group. Studies were classified as non-randomised if allocation concealment was viewed as inadequate [11]. Quasi-randomised studies were therefore classified as non-randomised. The intervention was categorised as NPWT if a medical device system identical or comparable to the vacuum-assisted closure (V.A.C.^®^) system was used. Studies were considered to be eligible only if publicly accessible full-text articles or other comprehensive study information (e.g. clinical study reports provided by manufacturers) were available.

For the present analysis, an identical and sufficiently large primary study pool, i.e. the pool of studies that could potentially be identified by all reviews, was required to ensure comparability between reviews. As a preliminary analysis showed that early reviews merely included 2 to 4 primary studies, only reviews published in or after December 2004 were considered.

Eligible reviews had to include data from completed primary studies on NPWT. Reviews were classified as systematic reviews (as opposed to narrative reviews) if multiple sources were searched (at least MEDLINE and The Cochrane Library), and the search strategy (including the search date) was documented [12].

Primary studies were eligible for inclusion only if they had been published before June 2004 and if the entry date of a study in a database preceded the date of the literature search of any systematic review analysed.

The methodology and primary study selection between reviews were compared, and the overall agreement in study selection between reviews was reported.

Only a summary of the reviews' quality assessment of primary studies and their conclusions on the effectiveness of NPWT is presented here, as the main focus of this paper was to compare the agreement in primary study selection between reviews.

## Results

**Figure 1:**
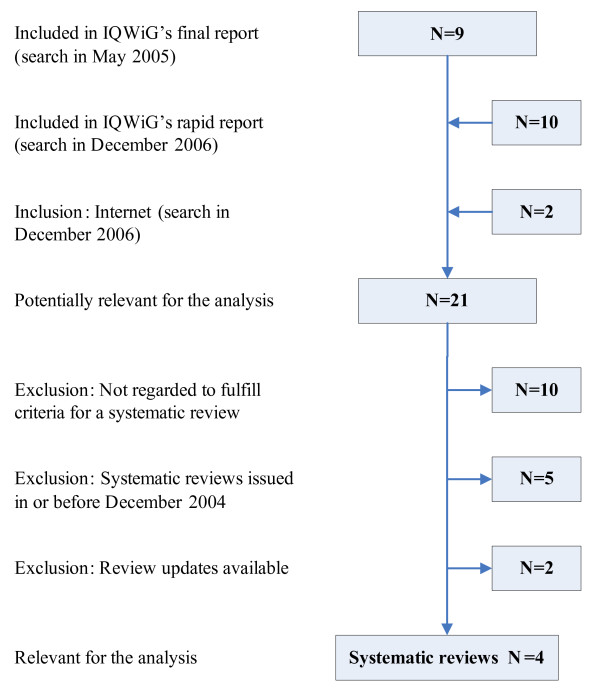
Flow chart of the review selection.

**Figure 2:**
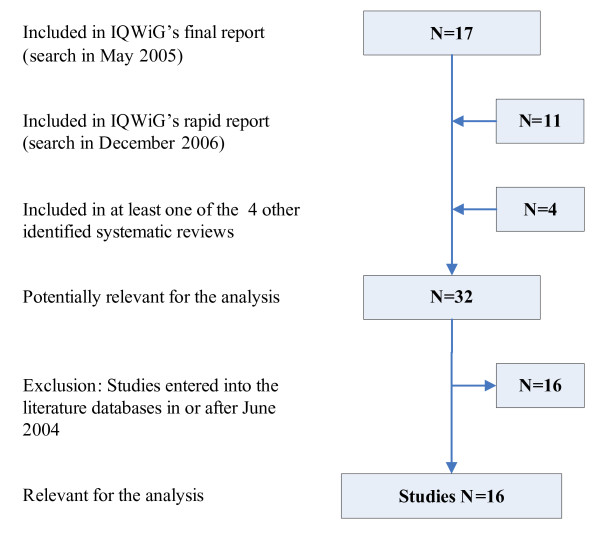
Flow chart of the study selection.

The flow charts of the selection of systematic reviews and primary studies are presented in Figures 1 and 2. Sixteen primary studies published before June 2004 were assessed in the present analysis [13-28]. A total of 5 eligible systematic reviews (the IQWiG review and 4 other systematic reviews) published between December 2004 and July 2006 were analysed [29-32]. Details on all reviews identified are shown in Table 1; the main reason for exclusion was failure to qualify as a systematic review.

**Table 1:** Identified pool of potentially relevant reviews

**No**	**Publication**	Documentation*	**Multiple sources**^†^	**Included**^‡^	**Reason for exclusion**
1	Andros 2006 [53]	-	-	-	Consensus statement
2	Brem 2006 [54]	-	-	-	Guidelines
3	Costa/MUHC TAU 2005 [30]^§^	+	+	+	-
4	Evans 2001 [55]	+	+	-	Systematic review published before 12/2004
5	Fleck 2006 [56]	-	-	-	Consensus statement
6	Fisher 2003 [57]	-	-	-	Narrative review
7	Gray 2004 [58]	+	+	-	Systematic review published before 12/2004
8	Hayes Inc. 2003 [59]			-	Not publicly accessible; published before 12/2004
9	Higgins 2003 [60]	+	+	-	Systematic review published before 12/2004
10	Mayer 2002 [61]	+	+	-	Not publicly accessible; published before 12/2004
11	Mendonca 2006 [62]	-	+	-	Search date not reported
12	OHTAC 2004 [63]	+	+	-	Update available: OHTAC 2006
13	OHTAC 2006 update [32]	+	+	+	-
14	Pham/ASERNIP-S 2003 [64]	+	+	-	Update available: Pham 2006
15	Pham/ASERNIP-S 2006 update [31]	+	+	+	-
16	Samson/AHRQ 2004 [29]	+	+	+	-
17	Shirakawa 2005 [65]	-	-	-	Narrative review
18	Suess 2006 [66]	-	-	-	Narrative review
19	Turina 2006 [67]	-	-	-	Narrative review
20	Ubbink 2006 [68]	-	-	-	Narrative review
21	Whelan 2005 [69]	-	-	-	Article on mechanisms of wound healing

*The literature search strategy was documented in detail (including search strategy and search date).

^†^Multiple sources used (at least MEDLINE and The Cochrane Library).

^‡^Criteria fulfilled: detailed documentation of the literature search; use of multiple sources; publication in or after December 2004.

^§^Costa 2007 [70] was not classified as an update, as no update of the literature search was performed.

AHRQ: Agency for Healthcare Research and Quality; ASERNIP-S: Australian Safety and Efficacy Register of New Interventional Procedures – Surgical; IQWiG: Institut für Qualität und Wirtschaftlichkeit im Gesundheitswesen (Institute for Quality and Efficiency in Health Care); MUHC TAU: McGill University Health Centre Technology Assessment Unit; NPWT: negative pressure wound therapy; OHTAC: Ontario Health Technology Advisory Committee.

The methods applied in the reviews included are presented in Tables 2 and 3. Regarding bibliographic databases, all reviews used MEDLINE, EMBASE, and The Cochrane Library, but the nursing database CINAHL was used only by IQWiG. The search terms applied varied between reviews. Regarding study design, the IQWiG review [8], as well as the reviews by Costa 2005 [30] and Pham 2006 [31] considered both RCTs and non-RCTs, while the reviews by Samson 2004 [29] and OHTAC 2006 [32] took only RCTs into account.

**Table 2:** Systematic reviews on NPWT: Requirements for primary studies and publications

**Systematic review**	**Interventions**	**Wound types**	**Clinical outcomes**	**Clinical study design**	**Sample size restrictions**	**Type of study information considered**	**Prespecified search for HTAs/SRs**	**Language restrictions**
**Samson/AHRQ 2004 **[29]	a) NPWT vs. other wound healing interventions b) NPWT + standard care vs. standard wound care alone c) NPWT vs. sham intervention	*Chronic wounds:* - pressure ulcers - metabolic disorders (e.g., diabetes mellitus) - vascular insufficiency - inflammatory disorders - malignancies - infections - miscellaneous (e.g., burns) *Other types of wounds:* - acute wounds - traumatic wounds - subacute wounds - dehisced wounds - partial thickness burns - diabetic ulcers - pressure ulcers - flaps - grafts	*Primary outcomes:* - incidence of complete wound closure - time to complete closure - adverse events *Secondary outcomes:* - facilitating surgical closure - need for debridement - infections - pain - activities of daily living - quality of life - improved cosmesis - change in wound size* - transcutaneous oxygen tension*	RCT	None	Articles	-	Abstracts published in English; articles without abstracts were reviewed if title indicated that articles met inclusion criteria; non-English articles were reviewed if English abstract indicated that articles met inclusion criteria.
**Costa/MUHC TAU 2005 **[30]^†^	NPWT vs. other treatment alternatives	Not prespecified in detail	Not prespecified in detail ("clinical effectiveness")	RCT Non-RCT (clinical comparative studies)	≥ 9 patients in either arm^‡^	Articles	+	Articles published in English or French
**IQWiG 2006 **[8]	NPWT vs. a) conventional wound therapy b) another type of NPWT	Acute or chronic wounds	- wound healing time - wound recurrence - revision operations - amputations - mortality - disease-related quality of life - activities of everyday life - pain - time spent in hospital - dressing changes - debridement procedures - adverse events - scar formation - subjective cosmetic results	RCT Non-RCT with a concurrent control group (clinical controlled trials, comparative cohort studies, case control studies)	None	Articles Unpublished data provided by manufacturers^§^	+	Language restrictions were not specified in the IQWiG review.^||^
**Pham/ASERNI P-S 2006 update **[31]^¶|^	NPWT vs. conventional methods	Particular wound types** - pressure ulcers and leg ulcers - diabetic foot ulcers and wounds - skin grafts - chronic wounds and complex/severe wounds - sternal wounds	Not prespecified in detail ("efficacy and safety outcomes")	RCT Non-RCT (other controlled or comparative studies and case series with consecutive patients)	None	Articles Conference abstracts^†† ^Manufacturer's information^††^	-	Searches were conducted without language restriction. English abstracts from non-English articles were included if they met the inclusion criteria and included efficacy and safety data.^‡‡^
**OHTAC 2006 (update) **[32]^§§^	NPWT vs. standard care	*Wounds, including* - Pressure ulcers - diabetic ulcers - sternal wounds - skin grafts	Not prespecified in detail ("Is negative pressure wound therapy effective for healing wounds...?")	RCT	≥ 20 patients	Articles ("peer-reviewed, published")	+	Articles published in English

*"Considered to be of less clinical importance" [29].

^†^Costa also considered economic outcomes.

^‡^One crossover study involving 7 patients was also included.

^§^Unpublished data from primary studies were only to be considered in the review if comprehensive study information (e.g. a clinical study report) was available.

^||^Additional information (IQWiG): An English-language title was required. No language restrictions were otherwise posed. If an English-language title or abstract indicated the potential relevance of a foreign-language text, the text was obtained and translated.

^¶^Personal communication (C. Perera, ASERNIP-S): "This publication draws from an accelerated systematic review which was published in 2003 and is accessible at . This review contains the full methodological details, including search strategies and inclusion/exclusion criteria. An accelerated systematic review uses the same methodology as a full systematic review, but may restrict the types of studies considered in order to produce the review in a shorter time period than the full systematic review. For example, accelerated reviews generally only include comparative studies and not case series, unless safety outcomes were inadequately described in the comparative evidence."

**Wound types listed in the results section, not in the methods.

^††^"Conference abstracts and manufacturer's information were included if they contained relevant safety and efficacy data." [31]

^‡‡^Information according to [31]. Additional information: "Searches for the review were conducted without language restriction in the first instance; however, included studies were limited to those published in English. An exception to this would be if there was a paucity of English language evidence, or if a landmark RCT was published in a non-English language, in which case the studies would then be translated and included." (Personal communication: C. Perera, ASERNIP-S).

^§§^OHTAC also considered economic outcomes.

AHRQ: Agency for Healthcare Research and Quality; ASERNIP-S: Australian Safety and Efficacy Register of New Interventional Procedures-Surgical; HTA: health technology assessment; IQWiG: Institut für Qualität und Wirtschaftlichkeit im Gesundheitswesen (Institute for Quality and Efficiency in Health Care); MUHC TAU: McGill University Health Centre Technology Assessment Unit; NPWT: negative pressure wound therapy; OHTAC: Ontario Health Technology Advisory Committee; RCT: randomised controlled trial; SR: systematic review.

**Table 3:** Systematic reviews on NPWT: Search strategies

**Systematic review**	**Search terms**	**Literature sources**					Search date*
			
		**MEDLINE**	**EMBASE**	**The Cochrane Library**	**CINAHL**	**Others**	
**Samson/AHRQ 2004 **[29]	*Vacuum-assisted closure:*^†^ - "topical negative pressure" - "sub-atmospheric pressure therapy" (also "subatmospheric") - "sub-atmospheric pressure dressing" (also "subatmospheric") - "vacuum sealing" - "vacuum assisted closure" - "negative pressure dressing" - "negative pressure therapy" - "foam suction dressing" - "vacuum compression" - "vacuum pack" - "sealed surface wound suction" - "sealing aspirative therapy" *Wounds:* - "wound*" - "ulcer*" - "decubit*" - "incision*" - "dressing" - "free flap" - "skin graft*" - "skin transplantation" - "degloving injuries" - "degloving injury"	+ MEDLINE via PubMed	+	+ CENTRAL	-	- Manufacturers^‡^	6/2004^§^

**Costa/MUHC TAU 2005**[30]	"vacuum" or "vacuum-assisted" or "VAC" or "negative pressure" or "suction dressing" or "subatmospheric" or "sub-atmospheric" or "subatmospheric pressure" or "NPWT" and "wound healing"^||^	+ PubMed	+	+ CDSR	-	- HTA agencies (CHSPR, MCHP, ICES, INAHTA) - Screening of reference lists of primary studies and secondary publications	3/2005

**IQWiG 2006 **[8]	Search strategies according to databases (published on pages 114 to 130 of the IQWiG review [8])	+ Ovid MEDLINE, Ovid MEDLINE In-Process and Other Non-Indexed Citations	+ Ovid EMBASE	+ CENTRAL; CDSR; DARE; HTA	+ Ovid CINAHL	- Trial registries (ClinicalTrials.gov, National Research Register) - Manufacturers^¶^ - Authorities - Conference proceedings - Authors of articles and conference abstracts - Screening of reference lists of secondary publications	5/2005

**Pham/ASERNIP-S 2006 update **[31]	(vacuum or suction) and (wound healing), (vacuum assisted or vacuum-assisted) and (wound or closure), topical negative pressure, (subatmospheric or sub-atmospheric) and pressure	+ MEDLINE; PREMEDLINE; PubMed	+	+ The Cochrane Library	-	- Current Contents - Trial registries (ClinicalTrials.gov, National Research Register) - The York (UK) Centre for Reviews and Dissemination - Grey literature reports - Relevant online journals - Vacuum therapy website (vacuumtherapy.co.uk) - The Internet	10/2004 New RCTs: 7/2005**

**OHTAC 2006 (update) **[32]	- wound healing - foot ulcer or skin ulcer or varicose ulcer or leg ulcer - wounds, non penetrating - chronic and ulcer or wound - leg or foot arterial or diabetic and ulcer or wound - suction - pressure - vacuum - vacuum assisted closure or V.A.C. therapy - negative pressure - topical negative pressure - subatmospheric pressure therapy	+ MEDLINE, MEDLINE In-Process and Non-Indexed Citations	+	+ CDSR	-	- HTA agencies (INAHTA) - Vacuum therapy website (vacuumtherapy.co.uk)	3/2006

*End of search period.

^† ^"The intersection of the vacuum-assisted closure terms and wound terms served as the initial pool of references. These were cross-referenced with the terms for randomized trials compiled by the Cochrane Collaboration...." For further details, please see Appendix A [29].

^‡^Request for "lists of published, randomized, controlled trials (RCTs), published abstracts of RCTs within the past 2 years, and published articles on study design, or protocols of any RCTs (published or in press)" [29].

^§^CENTRAL: 2003 ("through issue number 4", [29]).

^|| ^"Health technology agencies databases were also searched for technology assessment reports, systematic reviews and economic studies with the keywords 'vacuum', 'subatmospheric pressure', and 'sub-atmospheric pressure' used individually" [30].

^¶^Unpublished data from primary studies were only to be considered in the review if comprehensive study information (e.g. a clinical study report) was available.

** "Updated searches were performed in July 2005 to include any new RCTs" [31].

AHRQ: Agency for Healthcare Research and Quality; ASERNIP-S: Australian Safety and Efficacy Register of New Interventional Procedures – Surgical; CDSR: Cochrane Database of Systematic Reviews; CENTRAL: Cochrane Central Register of Controlled Trials; CHSPR: Centre for Health Services and Policy Research; DARE: Database of Abstracts of Reviews of Effects; CINAHL: Cumulative Index to Nursing and Allied Health Literature; EMBASE: Excerpta Medica Database; HTA: health technology assessment; CRD: Centre for Reviews and Dissemination; INAHTA: International Network of Agencies for Health Technology Assessment; IQWiG: Institut für Qualität und Wirtschaftlichkeit im Gesundheitswesen (Institute for Quality and Efficiency in Health Care); MCHP: Manitoba Centre for Health Policy; MUHC TAU: McGill University Health Centre Technology Assessment Unit; MEDLINE: Medical Literature Analysis and Retrieval System Online; NPWT: negative pressure wound therapy; OHTAC: Ontario Health Technology Advisory Committee; RCT: randomised controlled trial; VAC: vacuum-assisted closure.

As the comparison of systematic reviews based on published information showed numerous inconsistencies, we decided to contact the authors of the other reviews for clarification (this was not initially planned). We received responses from all authors approached (or from other researchers at the publishing institutions). After reviewing the responses, it became clear that reporting styles for excluded studies differed between reviews. For example, the response by OHTAC stated that "it must be noted that we do not routinely cite or analyse studies that have been excluded from our EBAs (evidence-based analyses)" [personal communication]. It consequently became apparent that some studies we had initially classified as "not identified by other reviews" had actually been identified but excluded, and subsequently not reported. We therefore changed the classification of studies not cited in reviews to "not reported". In addition, the authors of reviews corrected or clarified published information (their comments are included in Tables 4, 5, 6); in this context we thank them for generously providing information.

**Table 4:** Overview of primary study selection: comparison of trials included as RCTs by IQWiG

**Systematic review**	**Search date**	Primary study* (database entry date)^†^					**Personal communication with review authors or other researchers at the publishing institutions**
			
		**Joseph 2000 **[20]	**Ford 2002 **[17]	**Wanner 2003 **[27]	**Eginton 2003 **[16]	**Moues 2004 **[23]	
		(CIN: 2000-08; C)	(M: 2002-07-27; E; C)	(M: 2003-03-11; E; C)	(M: 2003-10-10; E; C)	(M: 2004-02-21; E; C; CIN)	
**Reviews considering RCTs and non-RCTs**							
**Costa/MUHC TAU 2005 **[30]	3/2005	RCT	RCT	RCT	RCT^‡^	RCT	**Eginton 2003**. *PC (V. Costa)*: "We classified the study as a crossover design. If I had to discern between RCT and non-RCT, I would consider it an RCT."

**IQWiG 2006 **[8]	5/2005	RCT	RCT	RCT	RCT	RCT	-

**Pham/ASERNIP-S 2006 update/**[31]	10/2004 (RCTs/non-RCTs) 7/2005 (RCTs)	RCT	RCT	RCT	RCT	RCT	-

**Reviews considering RCTs**							
**Samson/AHRQ 2004 **[29]	6/2004	RCT	RCT	RCT	RCT	RCT	-

**OHTAC 2006 **[32]	3/2006	RCT	RCT	RCT	Not reported (PC: excluded)	RCT	**Eginton 2003**. *PC (Medical Advisory Secretariat)*: "This citation was retrieved from the literature search, but was excluded as only 10 patients were enrolled. The exclusion was not documented in the HTA."

*Unless otherwise noted, the language of publication (abstract and full text) is English.

^†^Databases containing primary studies (entry date in database: yyyy-mm-dd).

^‡ ^"Crossover; first treatment selected randomly"[30].

AHRQ: Agency for Healthcare Research and Quality; ASERNIP-S: Australian Safety and Efficacy Register of New Interventional Procedures – Surgical; C: The Cochrane Library; CIN: CINAHL; E: EMBASE; HTA: health technology assessment; IQWiG: Institut für Qualität und Wirtschaftlichkeit im Gesundheitswesen (Institute for Quality and Efficiency in Health Care); M: MEDLINE; MUHC TAU: McGill University Health Centre Technology Assessment Unit; OHTAC: Ontario Health Technology Advisory Committee; PC: personal communication; RCT: randomised controlled trial.

**Table 5:** Overview of primary study selection: comparison of trials included as non-RCTs by IQWiG

**Systematic review**	**Search date**	Primary study* (Database entry date)^†^							**Personal communication with review authors or other researchers at the publishing institutions**
			
		**Genecov 1998 **[18]	**McCallon 2000 **[22]	**Doss 2002 **[15]	**Scherer 2002 **[24]	**Kamolz 2004 **[21]	**Schrank 2004 **[25]^‡^	**Wild 2004 **[28]^‡^	
		(M: 1998-04-02; E; C)	(M: 2001-02-24; C; CIN)	(M: 2002-12-07; E; C)	(M: 2002-07-31; E)	(M: 2004-04-15; E; CIN)	(M: 2004-05-29; E; C)	(M: 2004-05-29; E)	
**Reviews considering RCTs and non-RCTs**									
**Costa/MUHC TAU 2005 **[30]	3/2005	Non-RCT	Excluded: reason not stated in review (PC: sample size too small)	Non-RCT	Non-RCT	Not reported (PC: excluded)	Not reported (not applicable^§^)	Not reported (not applicable^§^)	**McCallon 2000**. *PC (V. Costa)*: "Did not meet our inclusion criteria (Appendix 1 of the report), i.e., < 9 patients per study arm. This study had 5 patients in each study arm." **Kamolz 2004**. *PC (V. Costa)*: "Did not meet our inclusion criteria, i.e., did not report clinical outcomes."

**IQWiG 2006 **[8]	5/2005	Non-RCT	Non-RCT: (allocation was based on alterna-tion)	Non-RCT	Non-RCT	Non-RCT	Non-RCT	Non-RCT	**Kamolz 2004**. IQWiG: The primary outcome was not a clinical but a surrogate outcome. "The perfusion of both hands was measured using the technique of dynamic laser-fluorescence-videography" [21]. IQWiG included this study because the outcome "pain" was reported in the results section ("All patients tolerated the V.A.C. application without major reports of pain and discomfort" [21]), although the method of pain measurement was unclear.

**Pham/ASERNIP-S 2006 update/**[31]^||^	10/2004 (RCTs/non-RCTs) 7/2005 (RCTs)	RCT	RCT	Non-RCT	Non-RCT	Not reported (PC: excluded)	Not reported (PC: excluded)	Not reported (PC: excluded)	**Genecov 1998**. *PC (C. Perera)*: "Allocation not stated, assumed that a valid method of randomisation had been utilised (critical appraisal in the full accelerated systematic review drew attention to this)." **McCallon 2000**. *PC (C. Perera)*: "As McCallon et al described this study as an RCT, the authors of the systematic review chose to classify it in the same way, despite the fact that patients were randomised based on alteration." **Kamolz 2004**. *PC (C. Perera)*: "Regarding the Kamolz study, treatment with VAC was focused upon the period immediately following trauma and thus these were not considered to be chronic, non-healing wounds which were the focus of this review and the manuscript." **Schrank 2004; Wild 2004**. *PC (C. Perera)*: "Regarding the Wild and Schrank studies, these were not published in English, however you are correct that these studies were published within the search dates. Had these studies been landmark RCTs, they would have been translated and included in the review."

**Reviews considering only RCTs**									
**Samson/AHRQ 2004 **[29]	6/2004	Excluded: no outcomes of interest (RCT)	RCT	Not reported (not applicable^¶^)	Not reported (not applicable^¶^)	Excluded, as the trial was a non-RCT	Not reported (not applicable^¶^)	Not reported (not applicable^¶^)	**Genecov 1998**. *PC (D. Samson)*: "This study was not a parallel groups or crossover randomized trial, but it was a within-subjects experimental design in which each participant served as his/her own control by receiving Opsite wound dressing and vacuum-assisted closure to separate wounds or wound areas. Since our review was focused on the primary outcome of progress to full wound healing and this study addressed only biopsy findings, this trial was excluded for reporting a non-relevant outcome." **McCallon 2000**. *PC (D. Samson)*: "Rather than excluding a marginal study like this based on quality concerns, our review selected an inclusive pool of randomized controlled trials, then evaluated study quality, noting that this trial ...used an allocation method that was probably inadequate to be considered true randomization (p. 57)."

**OHTAC 2006 **[32]	3/2006	Excluded: < 20 patients (RCT)	Excluded: < 20 patients (RCT)	Not reported (not applicable^¶^)	Not reported (not applicable^¶^)	Not reported (not applicable^¶^)	Not reported (not applicable^¶^)	Not reported (not applicable^¶^)	**Genecov 1998**. *PC (Medical Advisory Secretariat)*: "...the Genecov study is a case series of ten subjects and was incorrectly referred to as a randomized controlled trial (RCT). The study was excluded so how it was classified is not of particular relevance." **McCallon 2000**. *PC (Medical Advisory Secretariat)*: "... excluded based on the information reported in the abstract; there were less than 20 patients and the study was designated as an RCT by MEDLINE. We would not have retrieved the full text to further examine the study to determine how the randomization process was conducted given its exclusion based on number of subjects."

*Unless otherwise noted, the language of publication (abstract and full text) is English. For this analysis, all studies not classified as randomised trials in the systematic reviews were classified as non-RCTs by IQWiG.

^†^Databases containing primary studies (entry date: yyyy-mm-dd).

^‡^German full text.

^§^Review did not consider non-English or non-French full-text publications.

^||^Where authors had classified studies as RCTs, the studies were also classified as RCTs in the Pham review, regardless of the methods used to randomise patients. Where the method of randomisation was described, this was included in the critical appraisal section of the full systematic review, which is published on the ASERNIP-S website [personal communication: C. Perera, ASERNIP-S].

^¶^Review did not consider non-RCTs.

AHRQ: Agency for Healthcare Research and Quality; ASERNIP-S: Australian Safety and Efficacy Register of New Interventional Procedures – Surgical; C: The Cochrane Library; CIN: CINAHL; E: EMBASE; HTA: health technology assessment; IQWiG: Institut für Qualität und Wirtschaftlichkeit im Gesundheitswesen (Institute for Quality and Efficiency in Health Care); M: MEDLINE; MUHC TAU: McGill University Health Centre Technology Assessment Unit; OHTAC: Ontario Health Technology Advisory Committee; PC: personal communications; RCT: randomised controlled trial.

**Table 6:** Overview of primary study selection: comparison of trials excluded by IQWiG but included by at least one other review

**Systematic review**	**Search date**	Primary study* (Database entry date)^†^				**Personal communication with review authors or other researchers at the publishing institutions**
			
		**Davydov 1994 **[14]^‡^	**Catarino 2000 **[13]	**Song 2003 **[26]	**Jeschke 2004 **[19]	
		(M: 1994-09-01; C)	(M: 2001-01-13)	(M: 2002-12-24; E)	(M: 2004-02-06; E; C)	
**Reviews considering RCTs and non-RCTs**						
**Costa/MUHC TAU 2005 **[30]	3/2005	Not reported (not applicable^§^)	Non-RCT	Non-RCT	Not reported (PC: excluded)	**Jeschke 2004**. *PC (V. Costa)*: "Considered not eligible. Although negative pressure was used in one group, we considered that the main intervention studied was the Integra grafting and not vacuum-assisted closure. Moreover, since the group using VAC was a combined intervention (Integra + VAC), we were not sure if this would influence the results making it not an adequate estimate of the results with VAC alone. For these reasons the study was considered ineligible."

**IQWiG 2006 **[8]	5/2005	Excluded: not NPWT (non-RCT)^||^	Excluded: historical control (non-RCT)	Excluded: historical control (non-RCT)	Excluded: outcomes in the test group possibly affected by the additional intervention (RCT)	**Davydov 1994**. A translation of the full text of this Russian-language article showed that the intervention was not a technique comparable to NPWT. Consequently, IQWiG did not include this study. No reference to a randomised allocation was found. **Jeschke 2004**. NPWT was applied in combination with fibrin glue-anchored Integra in the test group receiving NPWT, but not in the control group receiving standard therapy; therefore the outcomes in the test group may have been affected by the additional intervention. Consequently, IQWiG did not include this study

**Pham/ASERNIP-S 2006 update/**[31]^¶^	10/2004 (RCTs/non-RCTs) 7/2005 (RCTs)	RCT	Non-RCT	Non-RCT	RCT	**Davydov 1994**. *PC (C. Perera)*: "Allocation not stated, assumed that a valid method of randomisation had been utilised (critical appraisal in the full accelerated systematic review drew attention to this)" **Jeschke 2004**. *PC (C. Perera)*: "Agree with IQWiG comments regarding additional intervention. Suggest that this RCT was included due to a paucity of RCT evidence on this indication (skin grafts), and as the other included RCT for skin grafts had only a 7 day follow up."

**Reviews considering only RCTs**						
**Samson/AHRQ 2004 **[29]**(6/2004; RCT)**	6/2004	Not reported (not applicable**)	Not reported (not applicable**)	Not reported (not applicable**)	Not reported (PC: not identified)	**Jeschke 2004**. *PC (D. Samson)*: "This study did not appear in our literature search, probably because it was not yet entered onto an electronic database by the date of our last search update."

**OHTAC **[32]**(3/2006; RCT)**	3/2006	Not reported (not applicable**)	Not reported (not applicable**)	Not reported (not applicable**)	Not reported (PC: excluded)	**Jeschke 2004**. *PC (Medical Advisory Secretariat)*: "...was included in the literature search results, but there were only twelve patients in the study so it was excluded. The exclusion was not documented in the HTA."

*Unless otherwise noted, the language of publication (abstract and full text) is English. For this analysis, all studies not classified as randomised trials in the systematic reviews were classified as non-RCTs by IQWiG. Studies not selected by any review are not listed in this table.

^†^Databases containing primary studies (entry date: yyyy-mm-dd).

^‡^Russian full text.

^§^Review did not consider non-English or non-French full-text publications.

^||^Study type not stated in the IQWiG review.

^¶^Where authors had classified studies as RCTs, the studies were also classified as RCTs in the Pham review, regardless of the methods used to randomise patients. Where the method of randomisation was described, this was included in the critical appraisal section of the full systematic review, which is published on the ASERNIP-S website [personal communication: C. Perera, ASERNIP-S]

**Review did not consider non-RCTs.

AHRQ: Agency for Healthcare Research and Quality; ASERNIP-S: Australian Safety and Efficacy Register of New Interventional Procedures – Surgical; C: The Cochrane Library; CIN: CINAHL; E: EMBASE; HTA: health technology assessment; IQWiG: Institut für Qualität und Wirtschaftlichkeit im Gesundheitswesen (Institute for Quality and Efficiency in Health Care); M: MEDLINE; MUHC TAU: McGill University Health Centre Technology Assessment Unit; NPWT: negative pressure wound therapy; OHTAC: Ontario Health Technology Advisory Committee; PC: personal communication; RCT: randomised controlled trial; VAC: vacuum-assisted closure.

Details of the primary study selection are presented according to the study classification by IQWiG in Tables 4 (5 RCTs), 5 (7 non-RCTs), and 6 (3 non-RCTs and 1 RCT excluded by IQWiG, but included by at least one other review).

The reviews included between 4 and 13 eligible primary studies published before June 2004. With regard to RCTs, the overall agreement in primary study selection between reviews was 96% (24 of 25 options) (Table 5).

More variations were noted concerning the selection of non-RCTs; the agreement between reviews considering both RCTs and non-RCTs was 57% (12 of 21 options). Of the 9 mismatches, according to published information and the information provided by authors or institutions, 7 were due to different inclusion criteria (e.g. language criteria), and 2 were due to variations in study classification (Table 5).

Four studies (3 non-RCTs and 1 RCT) were excluded by IQWiG but included by at least one other review. The reasons for exclusion were as follows: the study included historical controls (2 non-RCTs [13,26]); the intervention applied was not comparable to the NPWT technique (1 non-RCT [14]); or an additional intervention was applied that may have affected the study outcomes (1 RCT [19]) (Table 6). Substantial variations in study selection were shown between reviews.

Only the IQWiG review included a meta-analysis (changes in wound size), which indicated an advantage in favour of NPWT. However, only a few trials with small sample sizes were analysed.

The overall quality of the primary studies was assessed in 3 of 5 reviews, and was in general classified as poor. All reviews concluded that the evidence base on NPWT was insufficient (Table 7).

**Table 7:** Overall quality assessment of primary studies and main conclusions of systematic reviews on negative pressure wound therapy

**Systematic review**	Quality assessment conducted: yes/no and outcome*	**Main conclusion (direct quote)**
**Samson/AHRQ 2004 **[29]	Yes. 6× poor in quality	"The body of evidence is insufficient to support conclusions about the effectiveness of vacuum-assisted closure in the treatment of wounds."
**Costa/MUHC TAU 2005/**[30]	No	"Consequently, we agree with the conclusions of the previous technology assessment reports and systematic reviews [29,55,57,60,63,64] that there is insufficient evidence to recommend the routine use of this technology."
**IQWiG **[8]	Yes. 17× poor in quality	"There are at present no results of adequate reliability which provide proof of the superiority of NPWT in comparison with conventional therapy and which would justify broad use of this method outside clinical trial settings."
**Pham/ASERNIP-S 2006 update **[31]	No	"There is a paucity of high-quality RCTs on TNP for wound management with sufficient sample size and adequate power to detect any differences between TNP and standard dressings.".
**OHTAC **[32]	Yes. 1× moderate; 3× low; 2× very low overall quality	"Based on the evidence to date, the clinical effectiveness of NPWT to heal wounds is unclear."

*Numbers refer to all primary studies included in the reviews.

AHRQ: Agency for Healthcare Research and Quality; ASERNIP-S: Australian Safety and Efficacy Register of New Interventional Procedures – Surgical; HTA: health technology assessment; IQWiG: Institut für Qualität und Wirtschaftlichkeit im Gesundheitswesen (Institute for Quality and Efficiency in Health Care); MUHC TAU: McGill University Health Centre Technology Assessment Unit; NPWT: negative pressure wound therapy; OHTAC: Ontario Health Technology Advisory Committee; RCT: randomised controlled trial; TNP: topical negative pressure.

## Discussion

An analysis of 5 systematic reviews on NPWT showed differences (which mainly concerned non-RCTs) in the citation and selection of primary studies.

We would like to emphasize that by presenting these differences, we are not implying that the 4 other reviews identified were of inferior quality compared with the IQWiG review. Variations in the number of primary studies identified and selected are not surprising, as the reviews used different search strategies, literature sources, and inclusion criteria. After correspondence with the authors of the other reviews, many differences regarding the citation of primary studies could be attributed to different reporting styles (citation or non-citation) for excluded studies, not to the non-detection of studies in the literature searches.

Most differences in study selection resulted from variations in inclusion and exclusion criteria. For example, due to language restrictions, studies published in German were selected by IQWiG, but not by other reviews. Opinions on the relevance of language bias differ; a study published in 1997 comparing English and German-language publications concluded that English-language bias may be introduced in systematic reviews if they include only trials reported in English [33]. In contrast, a more recent publication noted that, for conventional medicinal interventions, language restrictions did not appear to bias estimates of effectiveness [34]. Moreover, for German-language publications on RCTs, it has been reported that German medical journals no longer play a role in the dissemination of trial results [35].

The inclusion criteria for primary study design were also inconsistent; 3 reviews (including the IQWiG review) considered both RCTs and non-RCTs, and 2 reviews considered only RCTs. The non-RCTs included in our analysis were non-randomised controlled intervention studies. However, there are many different study types that can be seen as non-RCTs (e.g., classical observational studies). The inclusion of non-RCTs in systematic reviews is inconsistent and controversial [36-40]. The validity of systematic reviews including non-RCTs may be affected by the differing susceptibility of RCTs and non-RCTs to selection bias [39], although it has been suggested that under certain conditions, estimates of effectiveness of non-RCTs may be valid if confounding is controlled for [40].

RCTs with adequately concealed allocation prevent selection bias and consequent distortions of treatment effects [41], and systematic reviews including RCTs represent the highest level of evidence for therapeutic interventions [42]. However, the quality and quantity of RCTs in surgical research is limited [43], and it has therefore been proposed not to base this type of research on RCTs alone [36,44]. Indeed, for some topics, non-RCTs are the only evidence available [45].

As for NPWT, although this treatment is widely applied in clinical practice, particularly in chronic wounds, at the time the IQWiG systematic review on NPWT was being planned only few RCTs were available; moreover, these were of poor quality [29]. However, there has been a recent increase in published RCTs, and as several of them are ongoing, more publications can be expected in the near future. One HTA agency has already changed its policy from including both RCTs and non-RCTs in systematic reviews on NPWT to one of including solely RCTs [32]. We agree with other researchers that non-RCTs should only be performed when RCTs are infeasible or unethical [38], and that systematic reviews including non-RCTs should only be conducted when RCTs are not available [39]. However, we emphasize that this should not be generalized to recommend excluding all kinds of non-randomised studies from systematic reviews on any topic and for any outcome of interest.

The type of non-RCT considered also differed: IQWiG's precondition for inclusion was the existence of a concurrent control group; studies with a historical control group were excluded, as systematic bias may arise from time trends in the outcomes of study participants [38].

Moreover, variations in the classification of study design were noted between reviews. For example, David Sampson, one of the other review authors, stated: "In general, our definition of randomized trials was probably more inclusive than yours. We decided to be inclusive due to the small number of potentially relevant studies available at that time. Our goal was to evaluate the quality of a larger pool of included studies rather than exclude more studies, based on quality concerns, to create a smaller pool of included studies" [personal communication].

As subjective factors are involved in the preparation of systematic reviews, inter-author variation is inevitable [46]. The evaluation of inter-author variation has shown that differences particularly affect the classification of study design [46,47]. One study showed that this was the case even when specific instructions and definitions were provided [47]. However, a recent analysis of the reproducibility of systematic reviews showed that, where authors were provided with guidelines for review preparation (including an algorithm to ensure that study designs were defined in a standardised manner), the overall reproducibility between reviews was good [48]. This finding emphasizes the relevance of standard reporting guidelines. The CONSORT statement on improving the quality of reporting for RCTs has been available for over a decade [49], and a revised version was published in 2001 [50]. In contrast, guidelines for non-RCTs are more recent [51,52]. The introduction of uniform reporting standards for non-RCTs may improve the future quality of reporting and lead to a closer agreement in the primary study citation and selection of systematic reviews.

Even though the reviews analysed included different numbers and types of studies, all reviews reached similar conclusions. This may be explained by the fact that the overall quality of the data on NPWT is poor.

## Conclusion

The citation and selection of primary studies differ between systematic reviews on NPWT, primarily with regard to non-RCTs. These differences arise from variations in review methodology and inter-author classification of study design, as well as from different reporting styles for excluded studies. Uniform methodological and reporting standards need to be applied to ensure comparability between reviews as well as the validity of their conclusions.

## Competing interests

The authors declare that they have no competing interests.

## Authors' contributions

StL and FP initiated the study. FP coordinated the study and conducted the literature search. StL, FP, and StS screened and analysed the retrievals. NM and FP drafted the manuscript. All authors interpreted the data and made an intellectual contribution to the manuscript. All authors reviewed and approved the final version.

## Pre-publication history

The pre-publication history for this paper can be accessed here:


